# Direct Medical Costs Associated With Post–COVID-19 Conditions Among Privately Insured Children and Adults

**DOI:** 10.5888/pcd20.220292

**Published:** 2023-02-09

**Authors:** Jamison Pike, Lyudmyla Kompaniyets, Megan C. Lindley, Sharon Saydah, Gabrielle Miller

**Affiliations:** 1 Immunization Services Division, National Center for Immunization and Respiratory Diseases, Centers for Disease Control and Prevention, Atlanta, Georgia; 2 National Center for Chronic Disease Prevention and Health Promotion, Centers for Disease Control and Prevention, Atlanta, Georgia; 3 National Center for Injury Prevention and Control, Centers for Disease Control and Prevention, Atlanta, Georgia

## Abstract

**Introduction:**

SARS-CoV-2, the virus that causes COVID-19, has caused more than 100.2 million infections and more than 1 million deaths in the US as of November 2022, yet information on the economic burden associated with post–COVID-19 conditions is lacking. We estimated the possible economic burden associated with post–COVID-19 conditions by comparing direct medical costs among patients younger than 65 years with and without COVID-19 in the postacute period.

**Methods:**

Commercially insured children and adults with a COVID-19 diagnosis (cases) during April–August 2020 were matched to those without COVID-19 (controls) on a 1:4 ratio. Direct medical costs represented 1-, 3-, and 6-month total expenditures per person starting 31 days after the diagnosis date. We used a 2-part model to evaluate cost differences among individuals with and without COVID-19, adjusted for patient characteristics.

**Results:**

Costs were higher among cases compared with controls. Direct medical costs among child cases were 1.82, 1.72, and 1.70 times higher than controls over 1, 3, and 6 months, respectively. Direct medical costs among adult cases were 1.69, 1.54, and 1.46 times higher than costs among controls over 1, 3, and 6 months, respectively. Relative differences in costs were highest among adults aged 50 to 64 years. In a subset of people with COVID-19, costs were higher among hospitalized cases compared with nonhospitalized cases.

**Conclusion:**

Our findings suggest a considerable economic burden of COVID-19 even after the resolution of acute illness, highlighting the importance of prevention and mitigation measures to reduce the economic impact of COVID-19 on the US health care system.

**Summary**

**What is already known on this topic?**

COVID-19 is associated with post–COVID-19 conditions, which can be a source of substantial health care costs.

**What is added by this report?**

To our knowledge, this is the first study to examine direct medical costs associated with post–COVID-19 conditions. We find significant excess medical costs associated with post–COVID-19 conditions during the first year of the pandemic.

**What are the implications for public health practice?**

Our findings suggest a considerable economic burden of COVID-19 even after the resolution of acute illness, highlighting the importance of prevention and mitigation measures to reduce the impact of COVID-19 on the US health care system and, ultimately, the US economy.

## Introduction

Approximately 100.2 million US residents have become infected, and more than 1 million have died of COVID-19 as of November 2022 (1). COVID-19 is associated with post–COVID-19 conditions (PCC), which can be a source of substantial health care costs (2). According to the Centers for Disease Control and Prevention (CDC), PCC are new, returning, or ongoing health problems that occur 4 or more weeks after first being infected with SARS-CoV-2, the virus that causes COVID-19, and can present as different types and combinations of health problems over different lengths of time (3). There is considerable uncertainty about the economic burden of PCC.

Previous studies have attempted to estimate short-term health care costs directly associated with an acute COVID-19 diagnosis (4–7). Using a large electronic administrative discharge database, Shrestha et al estimated a per-patient cost of $24,826 for inpatient care for adult patients with COVID-19. Tsai et al examined claims data and found that the mean cost per outpatient visit of a Medicare beneficiary with a COVID-19–related diagnosis was $164. Bartsch et al used simulation modeling and estimated median direct medical costs of a COVID-19 diagnosis ranging from $57 to $15,943, depending on the patient’s age and the severity of the case. Another study found that COVID-19–related hospital costs per adult hospitalization varied from $8,400 in a general ward to more than $50,000 in an intensive care unit with a ventilator (7). One study assessed out-of-pocket spending within 180 days after discharge from a COVID-19 hospitalization compared with patients hospitalized for pneumonia, aged 0 to 85 years or older. Across all age groups, they found that mean out-of-pocket spending after discharge for COVID-19 was $534 among privately insured patients and $680 among Medicare Advantage patients, compared with $445 and $918 for patients with pneumonia, respectively. However, none of these studies examined medical costs associated with PCC among children and adults.

Overall declines in health care spending during the pandemic may mask the economic burden of acute COVID-19 and PCC. Various factors have contributed to a decrease in health care use and spending in the US during the pandemic, including cancellations of elective care to increase hospital capacity and physical distancing measures to mitigate community spread of COVID-19. Whaley et al showed that substantial reductions in the use of preventive and elective care occurred during the first 2 months of the pandemic (8). A Commonwealth Fund report noted that reductions in outpatient care occurred across all types of specialties during the first 3 quarters of 2020 (with a mild rebound in May) (9). These reductions resulted in a drastic decline in health care spending (10,11). According to a Peterson-KFF Health System brief report, “As of December 2020, health services spending was down about 2.7% (seasonally adjusted annual rates) and it remained suppressed in January 2021” (11).

To estimate the possible health care–related economic burden of PCC, we used medical claims data to estimate post–COVID-19 direct medical costs of a patient younger than 65 years diagnosed with COVID-19 and then compared these costs to the costs of a patient not diagnosed with COVID-19. The results of this study can help quantify the true economic burden of COVID-19 and improve the accuracy of cost-effectiveness estimates of COVID-19 vaccination and other preventive measures.

## Methods

This study used data from IQVIA PharMetrics Plus database (12), a database of adjudicated medical claims from US health plans, including claims from primarily commercial health plans (preferred provider and health maintenance organizations), to provide a complete view of patient care across all care settings, including facility, management, surgical, ancillary, and pharmaceutical expenditures. We used the E360 Software-as-a-Service Platform (IQVIA) to extract IQVIA data (Native version, quarter 3 2021 data release). 

The sample included children (aged 0–17 y) and adults (aged 18–64 y) who were continuously enrolled in commercial health plans or self-insured employer groups from April 1, 2019, through March 31, 2021. Because our data source included only privately insured claims and did not capture Medicare claims, we did not include adults aged 65 years or older in our sample. Presence of a COVID-19 diagnosis was defined by using the *International Classification of Diseases, Tenth Revision, Clinical Modification* (ICD-10-CM) code of U07.1 (COVID-19, virus identified) from April 1, 2020, through August 31, 2020. Because COVID-19 vaccines were not available until December 2020, all cases and controls were unvaccinated at the time of diagnosis. Therefore, we were unable to directly assess the potential effect of vaccination on post–acute COVID-19 medical costs. Age was calculated as 2020 minus year of birth, because exact date of birth was not available.

We matched each person diagnosed with COVID-19 (cases) to 4 people without a COVID-19 diagnosis (controls) anytime during the study period. Controls may or may not have had health care encounters during the study period. We applied exact matching based on age, sex, insurance type, previous Charlson Comorbidity Index (CCI) (13,14), and number of previous hospitalizations. The CCI and number of hospitalizations were calculated by using diagnoses from April 1, 2019, through March 31, 2020.

The index date for cases was defined as the date of their first COVID-19 diagnosis; controls were assigned the index date of the case they were matched to. We identified patients with an index date during April 1–August 31, 2020. Direct medical costs represented total expenditures (paid by payer and patient) for each person during 1-, 3-, and 6-month periods starting 31 days after the index date. We excluded costs during 0 to 30 days after the index date, because these costs are more likely to reflect expenditures associated with acute illness rather than expenditures due to PCC. The study period was based on the most recent data available. Negative costs were censored at zero. We computed mean, median, and IQR of 1-, 3-, and 6-month cumulative direct medical costs among cases and controls, stratified by age category (children and adults).

To evaluate the difference in costs of patients, we used a 2-part model to account for the large number of observations with zero direct medical costs and the skewed distribution of nonzero costs. The probability that a case has nonzero costs is modeled in the first part by using a logistic regression. Estimates in the second part were generated by using a generalized linear model (GLM) with a log link and γ distribution.

We conducted analyses separately for children and adults. Models used cumulative 1-, 3-, or 6-month costs per person as the outcome and were adjusted for sex, age group (1–4, 5–11,12–15, and 16–17 y for children; 18–24, 25–39, 40–49, 50–64 y for adults), US census region, payer type (commercial, self-insured employer group, both), previous hospitalization from April 1, 2019, through March 31, 2020 (0, 1, ≥2), and previous CCI score calculated by using diagnoses from April 1, 2019, through March 31, 2020 (0, 1, ≥2 for children and 0, 1–2, 3–4, ≥5 for adults). The first set of models included presence of COVID-19 as the covariate of interest. The second set of models included a 3-way interaction between presence of COVID-19, sex, and age group, including lower-order interactions and main effects. The third set of models, restricted to individuals with COVID-19, included a presence of hospitalization with COVID-19 as the covariate of interest.

After each model, we estimated predicted costs among cases (P1) and among controls (P0). Excess costs were estimated in 2 ways: as the absolute differences in predicted costs (P1 minus P0) and relative differences in predicted costs (P1 divided by P0). All statistical analyses were performed in R version 4.2.1 (The R Foundation for Statistical Computing) and Stata version 15.1 (StataCorp LLC).

## Results

The analytic sample had 209,048 matched groups (197,412 adult cases; 11,636 child cases) (Table 1). The sample primarily comprised patients in the South and patients from self-insured employer groups, with a mean age of 12.1 years for children and 42.0 years for adults. Child cases had mean 1-, 3-, and 6-month costs of $434, $1,252, and $2,398, respectively, during the study period, while child controls had mean costs of $251, $761, and $1,434, respectively (Table 1). Adult cases had mean 1-, 3-, and 6-month costs of $957, $2,598, and $4,836, respectively, during the study period, while adult controls had mean costs of $560, $1,699, and $3,376, respectively (Table 1).

**Table 1:** Characteristics of Patients With COVID-19 (Cases) and Matched Patients Without COVID-19 (Controls), by Age Group and Time Period, and by Age Group and Sex, IQVIA, 2020–2021^a^

Characteristic	Children aged <18 years^b^		Adults aged 18–64 years^b^	
	Case	Control	Case	Control
**Total, no. (%)**	11,636 (100.0)	46,544 (100.0)	197,412 (100.0)	789,648 (100.0)
**Age, mean, y**	12.1	12.1	42.0	42.0
**Sex, no. (%)^a^**				
Female	5,938 (51.0)	23,752 (51.0)	104,361 (52.9)	417,444 (52.9)
Male	5,698 (49.0)	22,792 (49.0)	93,051 (47.1)	372,204 (47.1)
**Age, y, no. (%)**				
<5	1,255 (10.8)	5,020 (10.8)	—	—
5–11	3,035 (26.1)	12,140 (26.1)	—	—
12–15	3,568 (30.7)	14,272 (30.7)	—	—
16–17	3,778 (32.5)	15,112 (32.5)	—	—
18–24	—	—	30,540 (15.5)	122,160 (15.5)
25–39	—	—	51,121 (25.9)	204,484 (25.9)
40–49	—	—	45,354 (23.0)	181,416 (23.0)
50–64	—	—	70,397 (35.7)	281,588 (35.7)
**Insurance type, no. (%)**				
Commercial	3,880 (33.3)	15,520 (33.3)	70,001 (35.5)	280,004 (35.5)
Commercial and self-insured employer group	1,001 (8.6)	4,004 (8.6)	22,756 (11.5)	91,024 (11.5)
Self-insured employer group	6,755 (58.1)	27,020 (58.1)	104,655 (53.0)	418,620 (53.0)
**US census region, no. (%)**				
Northeast	906 (7.8)	7,465 (16.0)	19,859 (10.1)	132,856 (16.8)
Midwest	2,638 (22.7)	14,660 (31.5)	43,989 (22.3)	227,072 (28.8)
South	6,748 (58.0)	18,591 (39.9)	114,652 (58.1)	333,433 (42.2)
West	1,342 (11.5)	5,812 (12.5)	18,868 (9.6)	95,985 (12.2)
**No. of hospitalizations in previous year, no. (%)**				
0	11,370 (97.7)	45,480 (97.7)	188,391 (95.4)	753,564 (95.4)
1	228 (2.0)	912 (2.0)	7,765 (3.9)	31,060 (3.9)
≥2	32 (0.3)	128 (0.3)	1,042 (0.5)	4,168 (0.5)
**Charlson Comorbidity Index score in previous year, no. (%)**				
0	10,296 (88.5)	41,184 (88.5)	143,785 (72.8)	575,140 (72.8)
1	1,250 (10.7)	5,000 (10.7)	32,837 (16.6)	131,348 (16.6)
≥2	90 (0.8)	360 (0.8)	20,790 (10.5)	83,160 (10.5)
**Hospitalized with COVID-19**	207 (1.8)	—	18,559 (9.4)	—
**Costs, mean (median) [IQR], $**				
1-month	434 (0) [0–166]	251 (0) [0–91]	957 (37) [0–333]	560 (0) [0–196]
3-month	1,252 (164) [0–560]	761 (83) [0–380]	2,598 (325) [22–1,272]	1,699 (177) [0–778]
6-month	2,398 (401) [0–1,153]	1,434 (237) [0–729]	4,836 (822) [22–2,903]	3,376 (480) [0–1,851]
1-month (only ≥0 cost)	1,051 (227) [106–535]	758 (199) [93–440]	1,702 (273) [106–768]	1,216 (227) [83–619]
3-month (only ≥0 cost)	1,799 (350) [148–876]	1,252 (282) [125–679]	3,377 (588) [213–1,879]	2,523 (459) [172–1,432]
6-month (only ≥0 cost)	2,900 (565) [228–1,448]	1,926 (426) [181–1,054]	5,540 (1,108) [383–3,522]	4,291 (827) [292–2,659]

a Data source: IQVIA PharMetrics Plus (12), a database of adjudicated medical claims from US health plans, including claims from primarily commercial health plans (preferred provider and health maintenance organizations), to provide a complete view of patient care across all care settings, including facility, management, surgical, ancillary, and pharmaceutical expenditures.

b Some cells may not add up to 100% because of missing values or cell sizes <10.

In the 2-part model, we found that direct medical costs 31 or more days after the index date were higher among cases than controls (Table 2). Among children, excess medical costs during 1, 3, and 6 months were $208 (95% CI, $136–$281), $549 (95% CI, $361–$736), and $1,011 (95% CI, $717–$1,306), respectively. Medical costs were, on average, 1.75 times higher among children with COVID-19 than among children without COVID-19 during the 3 periods. Among adults, excess medical costs during 1, 3, and 6 months were $393 (95% CI, $359–$427), $930 (95% CI, $857–$1,003), and $1,562 (95% CI, $1,451–1,673), respectively. Medical costs were, on average, 1.56 times higher among adults with COVID-19 than among adults without COVID-19 during the 3 periods.

**Table 2:** Excess Direct Medical Cost Associated With Post–COVID-19 Conditions During 1, 3, and 6 Months Starting 31 Days After the Index Date, IQVIA 2020–2021^a^

Outcome	2-Part model for differences in cost between cases and controls (95% CI)^b^		*P* value^e^
	Absolute difference,^c^ $	Relative difference,^d^	
**Children**			
1-month	208 (136–281)	1.82 (1.52–2.20)	<.001
3-month	549 (361–736)	1.72 (1.46–2.02)	<.001
6-month	1,011 (717–1,306)	1.70 (1.49–1.94)	<.001
**Adults**			
1-month	393 (359–427)	1.69 (1.63–1.76)	<.001
3-month	930 (857–1,003)	1.54 (1.50–1.59)	<.001
6-month	1,562 (1,451–1,673)	1.46 (1.42–1.49)	<.001

a Data source: IQVIA PharMetrics Plus (12), a database of adjudicated medical claims from US health plans, including claims from primarily commercial health plans (preferred provider and health maintenance organizations), to provide a complete view of patient care across all care settings, including facility, management, surgical, ancillary, and pharmaceutical expenditures.

b Models were estimated separately among children and adults with the following outcome variables: 1-month cost, 3-month cost, and 6-month cost ≥31 days after the index date. All models include presence of COVID-19, sex, age group (1–4, 5–11, 12–15, and 16–17 y for children;18–24, 25–39, 40–49, 50–64 y for adults), US census region, payer type (commercial, self-insured employer group, both), previous number of hospitalizations (0, 1, ≥2) during April 1, 2019–March 31, 2020, and previous Charlson Comorbidity Index score (0, 1, ≥2 for children and 0, 1–2, 3–4, ≥5 for adults) during April 1, 2019–March 31, 2020.

c Calculated as costs among cases (individuals with a diagnosis of COVID-19) minus costs among controls (individuals without a diagnosis of COVID-19).

d Calculated as costs among cases (individuals with a diagnosis of COVID-19) divided by costs among controls (individuals without a diagnosis of COVID-19).

e Two-tailed *t* test with *P* < .05 for significance.

Excess costs of PCC among adults were highest among those aged 50 to 64 years (Table 3). For example, 6-month excess costs ranged from $892 (95% CI, $690–$1,094) for an adult aged 18 to 24 years to $2,191 (95% CI, $1,960–$2,421) for an adult aged 50 to 64 years. Excess 1-month and 3-month costs were significantly higher among men (vs women), whereas excess 6-month costs were significantly higher only for the relative difference in cost, not the absolute difference. Among children, excess costs were not significantly different by sex or age.

**Table 3:** Excess Direct Medical Cost Associated With Post–COVID-19 Conditions, by Age Group and Sex During 1, 3, and 6 Months Starting 31 Days After the Index Date, IQVIA 2020–2021^a^

Outcome	2-Part model for difference in cost between cases and controls^b^ (95% CI)					
	1-month		3-month		6-month	
	Absolute difference,^c^ $	Relative difference^d^	Absolute difference,^c^ $	Relative difference^d^	Absolute difference,^c^ $	Relative difference^d^
**Children**						
**By sex**						
Male	196 (101 to 291)	1.73 (1.35 to 2.21)	580 (313 to 847)	1.73 (1.35 to 2.21)	1,076 (631 to 1,522)	1.74 (1.43 to 2.12)
Female	215 (100 to 330)	1.90 (1.42 to 2.53)	430 (164 to 695)	1.55 (1.19 to 2.03)	821 (455 to 1,187)	1.56 (1.30 to 1.88)
**By age group, y**						
0–4	296 (−47 to 639)	2.25 (1.01 to 4.99)	61 (−674 to 795)	1.06 (0.51 to 2.19)	135 (−746 to 1,016)	1.08 (0.64 to 1.84)
5–11	214 (81 to 347)	2.38 (1.62 to 3.50)	628 (275 to 981)	2.37 (1.68 to 3.34)	1,074 (545 to 1,604)	2.21 (1.66 to 2.95)
12–15	183 (68 to 298)	1.64 (1.22 to 2.22)	574 (262 to 885)	1.72 (1.34 to 2.20)	992 (471 to 1,514)	1.66 (1.33 to 2.07)
16–17	185 (73 to 296)	1.59 (1.23 to 2.05)	512 (204 to 821)	1.56 (1.24 to 1.95)	1,123 (606 to 1,641)	1.63 (1.35 to 1.96)
**Adults**						
**By sex**						
Male	514 (444 to 583)	2.05 (1.90 to 2.20)	1,141 (988 to 1,293)	1.77 (1.67 to 1.88)	1,764 (1,551 to 1,977)	1.59 (1.52 to 1.67)
Female	329 (291 to 367)	1.53 (1.46 to 1.59)	848 (769 to 926)	1.45 (1.40 to 1.49)	1,530 (1,409 to 1,652)	1.41 (1.37 to 1.44)
**By age, y **						
18–24	177 (127 to 227)	1.44 (1.32 to 1.59)	478 (367 to 589)	1.42 (1.32 to 1.52)	892 (690 to 1,094)	1.39 (1.30 to 1.49)
25–39	290 (234 to 345)	1.58 (1.46 to 1.70)	698 (580 to 817)	1.46 (1.38 to 1.55)	1,185 (1,008 to 1,362)	1.40 (1.34 to 1.47)
40–49	378 (305 to 451)	1.68 (1.54 to 1.82)	787 (668 to 906)	1.46 (1.39 to 1.54)	1,398 (1,211 to 1,585)	1.42 (1.36 to 1.48)
50–64	570 (497 to 644)	1.89 (1.77 to 2.02)	1,375 (1,210 to 1,541)	1.70 (1.62 to 1.79)	2,191 (1,960 to 2,421)	1.56 (1.50 to 1.62)

a Data source: IQVIA PharMetrics Plus (12), a database of adjudicated medical claims from US health plans, including claims from primarily commercial health plans (preferred provider and health maintenance organizations), to provide a complete view of patient care across all care settings, including facility, management, surgical, ancillary, and pharmaceutical expenditures.

b Models were estimated separately among children and adults with the following outcome variables: 1-month cost, 3-month cost, and 6-month cost ≥31 days after the index date. All models included the interaction of presence of COVID-19, sex, and age group (1–4, 5–11,12–15, and 16–17 y for children;18–24, 25–39, 40–49, 50–64 y for adults), including lower-order interactions and main effects, US census region, payer type (commercial, self-insured employer group, both), previous number of hospitalizations (0, 1, ≥2) during April 1, 2019–March 31, 2020, and previous Charlson Comorbidity Index (0, 1, ≥2 for children and 0, 1–2, 3–4, ≥5 for adults) during April 1, 2019–March 31, 2020.

c Calculated as costs among cases (individuals with a diagnosis of COVID-19) minus costs among controls (individuals without a diagnosis of COVID-19).

d Calculated as costs among cases (individuals with a diagnosis of COVID-19) divided by costs among controls (individuals without a diagnosis of COVID-19).

In a subset restricted to individuals with a COVID-19 diagnosis, we found direct medical costs were higher among those with a COVID-19 hospitalization, compared with those without (Table 4). Among children, medical costs were 15.64, 12.29, and 9.47 times higher during the 1-, 3-, and 6-months, respectively, for those with COVID-19 hospitalization, compared with those without. Among adults with a COVID-19 diagnosis, medical costs were 5.18, 3.95, and 3.18 times higher during the 1-, 3-, and 6-months, respectively, for those with COVID-19 hospitalization compared with those without.

**Table 4:** Differences in Direct Medical Cost Among Patients With Inpatient vs Outpatient COVID-19 Starting 31 Days After the Index Date, in a Subset of Patients With COVID-19, IQVIA 2020–2021^a^

Outcome	2-Part model for differences in cost between cases and controls (95% CI)^b^	
	Absolute difference,^c^ $	Relative difference^d^
**Children**		
1-month	5,043 (2,392–7,693)	15.64 (9.47–25.84)
3-month	11,776 (4,927–18,625)	12.29 (7.17–21.05)
6-month	17,706 (7,659–27,753)	9.47 (5.68–15.79)
**Adults**		
1-month	2,676 (2,406–2,946)	5.18 (4.74–5.66)
3-month	5,711 (5,128–6,294)	3.95 (3.65–4.29)
6-montht	8,412 (7,606–9,218)	3.18 (2.97–3.41)

a Data source: IQVIA PharMetrics Plus (12), a database of adjudicated medical claims from US health plans, including claims from primarily commercial health plans (preferred provider and health maintenance organizations), to provide a complete view of patient care across all care settings, including facility, management, surgical, ancillary, and pharmaceutical expenditures.

b Analysis was restricted to patients with COVID-19 only. Models were estimated separately among children and adults with COVID-19 with the following outcome variables: 1-month cost, 3-month cost, and 6-month cost ≥31 days after the index date. All models include presence of a hospitalization with COVID-19, sex, age group (1–4, 5–11,12–15, and 16–17 y for children;18–24, 25–39, 40–49, 50–64 y for adults), US census region, payer type (commercial, self-insured employer group, both), previous number of hospitalizations (0, 1, ≥2) during April 1, 2019–March 31, 2020, and previous Charlson Comorbidity Index score (0, 1, ≥2 for children and 0, 1–2, 3–4, ≥5 for adults) during April 1, 2019–March 31, 2020.

c Calculated as costs among cases (individuals with a diagnosis of COVID-19) minus costs among controls (individuals without a diagnosis of COVID-19).

d Calculated as costs among cases (individuals with a diagnosis of COVID-19) divided by costs among controls (individuals without a diagnosis of COVID-19).

## Discussion

To our knowledge, our study is the first to examine potential direct medical costs associated with PCC. We demonstrated a notable difference in direct medical costs among both children and adults following a COVID-19 diagnosis compared with patients not diagnosed with COVID-19. Specifically, average excess medical costs were 70% higher for children and 46% higher for adults during the 6-month period; application of these costs across our cohort during the study period results in an additional economic burden of up to $47 million for children and up to $308 million for adults in these cohorts.

The results of this study provide valuable insight into the economic burden of PCC. All cases in this study were unvaccinated when they were diagnosed with COVID-19. Therefore, this analysis can be considered an estimate of the excess medical costs associated with PCC in an unvaccinated patient.

Men and older adults in our sample had higher excess costs associated with PCC. A previous study showed that older individuals were more likely to experience incident postacute sequelae (15). Exactly why men have higher excess costs than women is unknown. Studies have shown that men are less likely than women to have PCC (16); this is supported by our data, which showed that 43% of men and 57% of women with COVID-19 had positive (non-zero) 6-month costs. Despite having fewer positive costs, PCC costs were higher among men than among women. This could be, in part, because of men having more acute COVID-19 illness (17), which is known to be associated with a higher incidence of PCC (15). Previous studies also showed differences by sex in the manifestation of PCC: women were more likely to experience ear, nose, and throat problems, whereas men were more likely to have diabetes or kidney disease (18).

Considerable uncertainty exists about the economic burden of PCC. PCC are continuously being identified and studied. In early 2021, with $1.15 billion in support from Congress, the National Institutes of Health announced an initiative to identify the causes and ultimately the means of preventing and treating individuals with PCC (19), and as of July 2021, PCC can be considered a disability under the Americans with Disabilities Act (3). PCC are now identified by using the general ICD-10-CM code U09.9 (post COVID-19 condition, unspecified); however, this code went into effect in October 2021, after our study period ended (20). Even without a specific diagnosis code to identify cases of PCC, our methodology captures patients with likely PCC and can be considered a lower bound of the additional health care utilization cost of a PCC diagnosis. A recent study of worker’s compensation claims associated with COVID-19 suggests substantial medical costs are attributable to the small proportion of patients with prolonged disability following COVID-19 diagnosis, further highlighting the potential economic impact of PCC (21).

Our findings also lay the foundation for further economic analysis. The economic costs associated with PCC estimated in this study include only direct medical costs, which represent payments made to providers. We did not estimate the difference in the economic burden of out-of-pocket costs of a patient after a COVID-19 diagnosis, nor could we capture the difference in productivity losses of patients or caregivers. Studies evaluating these additional costs would improve the understanding of the full economic ramifications of the COVID-19 pandemic and could better inform cost-effectiveness or cost-benefit analyses that examine the impact of various prevention and control strategies. Additionally, future studies may examine long-term direct medical costs associated with COVID-19 among vaccinated individuals and those with underlying medical conditions associated with an increased risk of severe COVID-19 illness.

### Limitations

Some limitations apply to these findings. First, the ICD-10-CM code for COVID-19 diagnosis was introduced in April 2020, so some of our controls may have had COVID-19 previously. Second, lower-income and racial and ethnic minority groups were disproportionately affected early in the pandemic; however, our data source did not capture data on socioeconomic, racial, or ethnic characteristics, which could further highlight cost differences. Third, this analysis captures health care costs for PCC only during the first year of the pandemic, before the emergence of variants such as Delta and Omicron, which may have different cost profiles. Furthermore, because not everyone with COVID-19 receives a diagnosis through a medical encounter (because of lack of access to testing or to health care services generally or through failure to seek care for mild or asymptomatic cases), cases in our sample may not represent the average case, and the control group may include undiagnosed cases. Because no diagnosis code for PCC existed during the study period, we were unable to identify potential undiagnosed COVID-19 cases in our control group; such cases could potentially bias our findings downward. Our case cohort would not capture individuals with COVID-19 who died before having a health care encounter. Finally, our data source included privately insured persons younger than 65 years, so our results may not be generalizable to older adults or persons who are publicly insured or uninsured.

Using a large, geographically dispersed database of adjudicated medical claims while controlling for a wide range of factors, we found significant excess medical costs associated with PCC during the first year of the pandemic. Overall, our findings suggest a considerable economic burden of COVID-19 even after the resolution of acute illness, highlighting the importance of mitigation measures to reduce the impact of COVID-19 on the US health care system and ultimately, the US economy.
